# CRISPR Interference Efficiently Silences Latent and Lytic Viral Genes in Kaposi’s Sarcoma-Associated Herpesvirus-Infected Cells

**DOI:** 10.3390/v13050783

**Published:** 2021-04-28

**Authors:** Kevin Brackett, Ameera Mungale, Mary Lopez-Isidro, Duncan A. Proctor, Guillermo Najarro, Carolina Arias

**Affiliations:** 1 Department of Molecular, Cellular, and Developmental Biology, University of California, Santa Barbara, CA 93106, USA; kevinlbrackett@ucsb.edu (K.B.); ameeramungale@umail.ucsb.edu (A.M.); mlopezisidro@umail.ucsb.edu (M.L.-I.); duncan@ucsb.edu (D.A.P.); gnajarro@ucsb.edu (G.N.); 2 Neuroscience Research Institute, University of California, Santa Barbara, CA 93106, USA; 3 Center for Stem Cell Biology and Engineering, University of California, Santa Barbara, CA 93106, USA

**Keywords:** KSHV, CRISPR-interference, dCas9-KRAB, Kaposi’s sarcoma-associated herpesvirus, gene expression, gene silencing

## Abstract

Uncovering viral gene functions requires the modulation of gene expression through overexpression or loss-of-function. CRISPR interference (CRISPRi), a modification of the CRISPR-Cas9 gene editing technology, allows specific and efficient transcriptional silencing without genetic ablation. CRISPRi has been used to silence eukaryotic and prokaryotic genes at the single-gene and genome-wide levels. Here, we report the use of CRISPRi to silence latent and lytic viral genes, with an efficiency of ~80–90%, in epithelial and B-cells carrying multiple copies of the Kaposi’s sarcoma-associated herpesvirus (KSHV) genome. Our results validate CRISPRi for the analysis of KSHV viral elements, providing a functional genomics tool for studying virus–host interactions.

## 1. Introduction

Kaposi’s sarcoma-associated herpesvirus (KSHV) is an oncogenic gammaherpesvirus associated with the development of Kaposi’s sarcoma (KS) and two lymphoproliferative disorders, primary effusion lymphoma (PEL) and multicentric Castleman’s disease (MCD), in immunocompromised patients [1]. KSHV is endemic to Sub-Saharan Africa, where it remains a prevalent medical problem, with ~40 thousand cases of KS each year, accounting for approximately 20% of all cancers in the region (fifth most common cancer) [2,3]. In the United States, 1 in 200 transplant patients develop pathologies associated with KSHV reactivation, leading to transplant rejection or death [4].

KSHV has a large, circular dsDNA genome of 160–170 Kb, which encodes over 90 open reading frames (ORFs), more than two dozen short ORFs and upstream ORFs, circular RNAs, several long noncoding RNAs (ncRNAs), and 25 micro RNAs [5,6]. Functional genomics studies have revealed the role of several viral ORFs and ncRNAs in immunomodulation [7], oncogenesis [3,8], and the basic biology of the abduction of the cellular machinery by the virus [9,10,11,12,13]. Nevertheless, several viral elements’ biological roles, including long ncRNAs and alternative transcripts, remain unclear. The generation of loss-of-function mutants is the most effective strategy for the functional characterization of viral genes. The construction of KSHV bacterial artificial chromosome (BAC) clones and recombineering techniques have propelled the systematic analysis of KSHV gene functions [14,15]. However, recombineering KSHV BACs remains a time-consuming and often challenging endeavor, typically restricted to one or a few genes.

Post-transcriptional gene silencing and genome editing through RNA interference (RNAi) and CRISPR-Cas9, respectively, have expanded the functional genomics toolkit to study KSHV genes’ function and virus–host interactions [9,16,17,18,19,20]. RNAi using small interfering RNAs (siRNAs) and short hairpin RNAs (shRNAs) has been useful for depleting many KSHV transcripts. However, RNAi has inherent limitations, including off-target effects, the competition with endogenous substrates for the cell’s RNAi machinery, and their inability to target nuclear transcripts [21,22,23]. CRISPR editing overcomes these limitations and has been recently used to generate single viral gene knockouts or as part of genetic screens in KSHV-infected cells [16,17,18,24]. Nevertheless, CRISPR editing is encumbered by the viral genome multiplicity naturally observed in KSHV-infected cells, ranging from a handful to over 100 viral genomes per cell [25]. Consequently, CRISPR editing of viral loci present in an infected cell can be a mammoth undertaking, requiring the screening and selection of single cells carrying mutations in every KSHV episome [24]. Moreover, even under stringent selection conditions, it is still difficult to ensure that every viral genome has been edited to carry the same mutation.

CRISPR interference (CRISPRi) offers an alternative approach. CRISPRi represses gene expression using a catalytically dead Cas9 (dCas9) fused to the transcriptional repressor domain Krüppel-associated box (KRAB), recruited to gene promoters or open reading frames by sgRNAs [26,27]. Upon binding to the target DNA sequence, the dCas9-KRAB complex (dC9K) represses transcription initiation or elongation by steric hindrance [26]. Repression is enhanced by the KRAB domain-mediated recruitment of heterochromatin-forming factors, which epigenetically silence the gene [28]. Unlike CRISPR-editing, CRISPRi does not require the generation of individual mutations at single loci to achieve a close to complete loss-of-function phenotypes in mammalian, yeast, and bacterial cells [29,30,31]. This characteristic makes CRISPRi an ideal technology to carry out functional genomic studies in virus-infected cells where the simultaneous targeting of multiple individual episomes is required for effective viral gene silencing. Here, we report the development of a CRISPRi system to silence viral genes in KSHV-infected epithelial and B-cells. We show that CRISPRi efficiently represses latent and lytic KSHV genes and that it is unfettered by the numerous viral genomes present in infected cells. CRISPRi complements traditional mutagenesis approaches and offers a straightforward and rapid alternative for the interrogation and characterization of KSHV gene functions.

## 2. Materials and Methods

### 2.1. Cell Culture

iSLK.219 and HEK 293METR cells were grown in Dulbecco’s modified Eagle medium (DMEM; Invitrogen, Carlsbad, CA, USA) supplemented with 10% FBS, 200 uM of L-glutamine, and 100 U/mL of penicillin and streptomycin. iSLK-219 cells were maintained in 10 μg/mL of puromycin (Invivogen, San Diego, CA, USA). BCBL-1 were grown in RPMI 1640 medium (Invitrogen, Carlsbad, CA, USA) supplemented with 10% fetal bovine serum (FBS; Invitrogen, Carlsbad, CA, USA), 200 μM of L-glutamine, and 100 U/mL of penicillin/streptomycin. Exogenous RTA expression was induced in iSLK-219 cells by treatment with 1 μg/mL of doxycycline (Fisher Scientific, Waltham, MA, USA).

### 2.2. Generation of iSLK-219-dC9K and BCBL-1-dC9K

VSV-G pseudotyped lentiviruses for the generation of cell lines expressing dCas9-BFP-KRAB (IGI-p0165 generous gift of the Innovative Genomics Institute, Berkeley, CA 94704, USA) were produced using standard protocols in HEK293-METR cells [32]. Lentiviral supernatants were collected in virus collection medium (DMEM containing 4.5 g/L glucose supplemented with 4–10% FBS, 15 mM HEPES, L-glutamine, sodium pyruvate and penicillin/streptomycin) and concentrated using regenerated cellulose centrifugal filter units with a 100 k MW cut-off (Amicon Ultracel 100 k/3000 rpm/10 min). The concentrated lentiviral supernatant was used to infect iSLK-219 or BCBL-1 cells by spinoculation (2000 rpm/2 h/RT) in 6 well plates. The cells were allowed to recover for 24 h and were selected with blasticidin (10 μg/mL) for 10 days. The cells were then sorted for BFP expression in the FACSAria II sorter (BD).

### 2.3. sgRNA Design and Transduction

sgRNAs for CRISPRi of KSHV (accession number GQ994935.1) were designed as described by Horlbeck et al. [33] (Table S1). Synthetic DNA segments encoding the sgRNAs were cloned into the pHR-SFFV-dCas9-BFP-KRAB (Addgene 46911) at BstXI and XhoI, and the clones were confirmed by sanger sequencing. Lentiviral production and transduction were performed as described above. After transduction, BCBL-1 cells were maintained in 1 μg/mL of puromycin for 10 days prior to the selection of BFP+/sgRNA+ by FACS in a Sony SH800 instrument. iSLK-219 cells were selected for BFP+/sgRNA+ expression by FACS in a Sony SH800 instrument (see Tables S1 and S2 for sgRNA sequences and genomic coordinates).

### 2.4. Immunoblotting and Antibodies

Cells were washed and collected in 1× sample buffer (62.5 mM Tris-HCl (pH 6.8), 2% sodium dodecyl sulfate (SDS), 10% glycerol, 0.7 M β-mercaptoethanol). Cell lysates were fractionated by SDS-PAGE and transferred onto nitrocellulose membranes. Immunoblots were incubated with primary antibodies overnight at 4 °C and immunoreactive bands were detected with HRP-conjugated secondary antibodies by enhanced chemiluminescence (ThermoFisher, Waltham, MA, USA) according to the manufacturer’s recommendations. All antibodies were used at a 1:1000 dilution in 3% BSA/1× TBST, unless indicated. Antibodies: ORF57 (SBCT sc-135746), ORF59 (Advanced Biotechnology 13-211-100), PERK (Cell Signaling 5683), bActin (1:30,000, Sigma Aldrich, St. Louis, MO, USA), GAPDH (1:20,000 Abcam, Cambridge, UK), Vinculin (Cell Signaling 4650), K8.1 (mAb clone 19B4) [34]. The LANA rabbit polyclonal antibody was raised against a synthetic peptide from the acidic domain of LANA (Polson and Ganem, unpublished).

### 2.5. Immunofluorescence

Cells were fixed in 4% paraformaldehyde (PFA) at room temperature for ten minutes, or in 90% ice-cold methanol as indicated. Fixed cells were washed with 1× PBS and blocked in 3% BSA/1× PBS/0.1% Triton, at room temperature for 1 h. Cells were incubated with 1:1000 Rabbit anti-LANA antibody overnight at 4 °C. Cells were washed with PBS- 0.1% Triton (PBST) and incubated with a secondary antibody (anti-Rabbit Alexa 488, 1:2500) and DAPI, for 1 h at room temperature. Cells were washed with PBST and then imaged using the LEICA SP8 confocal microscope.

### 2.6. Quantitative Reverse Transcription PCR

Cells were lysed with RLT Lysis Buffer and RNA was extracted using an RNeasy Mini Kit (Qiagen, Hilden, Germany). cDNA synthesis was performed using iScript cDNA Synthesis Kit (Bio-Rad) following the manufacturer’s recommendations. Quantitative polymerase chain reaction (qPCR) was performed in triplicate using the 2× SYBR green mix (ThermoFisher, Waltham, MA, USA). Primer sequences for ATF6 Fwd-5′-CCTGCTGTTACCAGCTACCAC-3′, Rev-5′-CCAAAGAAGGTGTTGGTTTGA-3′; primer sequences for 28S Fwd-5′-AAACTCTGGTGGAGGTCCGT-3′, Rev-5′-CTTACCAAAAGTGGCCCACTA-3′.

### 2.7. Virus Tittering

The viral titer was determined by collecting lytic cells’ supernatants that were clarified, filtered with a 0.45 um syringe filter, and diluted before the spinoculation (2000 rpm/2 h/Room temp) of uninfected iSLK in 6 well plates. Cells were incubated for 48 h, trypsinized and collected for flow cytometry in a Sony SH800 instrument. The percentage of cells expressing EGFP was determined by flow cytometry and used to calculate the number of fluorescence forming units (ffus) in each sample.

## 3. Results

### 3.1. CRISPRi Represses Viral Genes in KSHV Infected Cells

**Figure 1:**
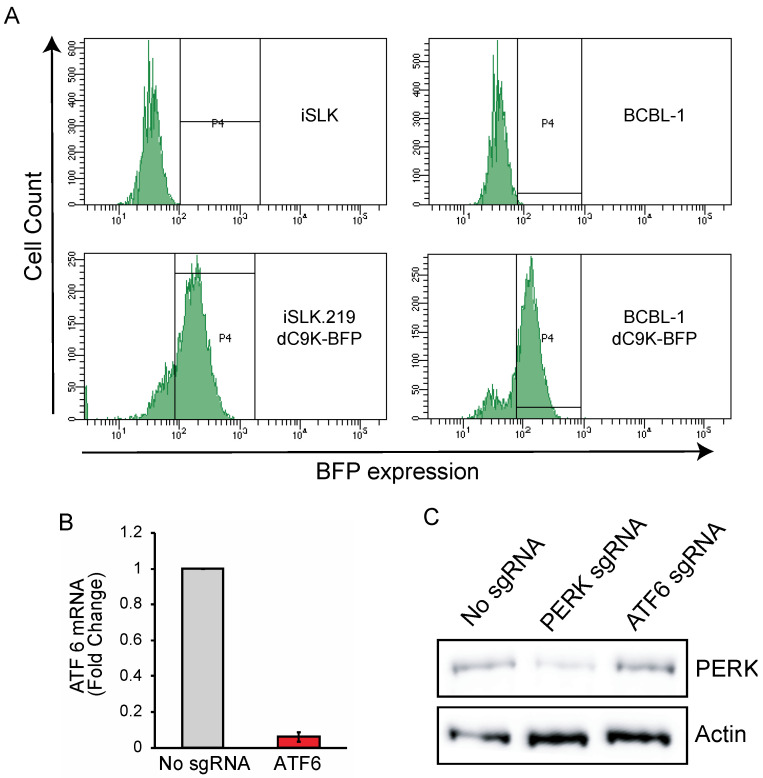
Generation of iSLK-219-dC9K and BCBL-1-dC9K cell lines. (**A**) FACS traces showing mean fluorescence intensity (MFI) of iSLK-219 and BCBL-1 cells transduced with a lentiviral vector expressing dCas9-KRAB-BFP-Blasticidin, selected with blasticidin for 10 days and sorted by FACS for BFP+ expression (P4 gate) (**B**) RT-qPCR showing silencing of ATF6 in BCBL-1 cells. BCBL-1-dC9K cells were transduced with an ATF6 sgRNA targeting vector and selected with puromycin for 10 days. 28S rRNA was used as a loading control. Error bars: standard deviation (**C**) immunoblot showing silencing of PERK 10 days after iSLK-219 cells were transduced with a PERK sgRNA targeting vector and selected by FACS (BFP+).

To silence viral genes using CRISPRi, we engineered two KSHV latently infected cell lines, iSLK-219 and BCBL-1, to constitutively and stably express dCas9-KRAB (dC9K) (Figure 1A). The epithelial cell line iSLK-219, and the B-cell line BCBL-1 are robust, commonly used KSHV infection models [35,36]. iSLK-219s are latently infected with the recombinant KSHV.219 strain, which encodes constitutive (EGFP) and lytic-reactivation-inducible (RFP) reporters [37]. These cells also harbor a doxycycline (Dox) inducible transgene encoding the viral transcription factor RTA (replication and transcriptional activator), which acts as the master switch for the transition from the latent to the lytic stages of infection [38]. BCBL-1 cells are derived from a PEL patient and are naturally infected with wild-type KSHV [35]. To generate CRISPRi KSHV-infected cells, we transduced iSLK-219 and BCBL-1 cells with lentiviruses encoding dC9K fused to a blue fluorescent protein (BFP). Initially, we attempted to establish iSLK-219 cell lines that stably express dC9K but noted that the transgene was lost after transduction (data not shown). We used a lentiviral vector with a blasticidin resistance marker to overcome this limitation and maintained the transgene for several generations under blasticidin selection. Finally, we selected pseudoclonal cell populations of high dC9K expression by fluorescence-activated cell sorting (FACS) and maintained the sorted cells under blasticidin selection (Figure 1A).

To test whether the CRISPRi system was functional in the cell lines mentioned above, we transduced them with sgRNAs targeting the promoters of the endogenous genes PERK and ATF6, which encode ubiquitously expressed endoplasmic reticulum stress sensor proteins (Table S1) [39]. The lentiviral vector encoding the sgRNAs also encodes BFP and a puromycin selection marker, allowing the pharmacological and FACS-based selection of positive transductants. We selected BCBL-1-dC9K cells expressing the ATF6 targeting sgRNAs by treating them with puromycin. Next, we measured the expression levels of ATF6 by RT-qPCR and observed robust repression (>90%) of the corresponding mRNA in cells expressing the targeting sgRNA (Figure 1B). As the KSHV.219 recombinant virus present in iSLK-219-dC9K has a puromycin resistance marker, we selected BFP^+^ cells expressing the PERK targeting sgRNA by FACS. The expression of BFP is more efficient from the sgRNA vector than from the dCas9-KRAB vector, thus allowing for the selection of sgRNA transduced cells based on BFP^+^ status. We evaluated the efficiency of PERK silencing by CRISPRi in the iSLK-219-dC9K cell by immunoblot and observed a substantial reduction in PERK protein levels (Figure 1C). Together, these results show that, expectedly, CRISPRi efficiently silences host gene promoters in KSHV-infected iSLK.219 and BCBL-1 cells (Figure 1B,C).

**Figure 2:**
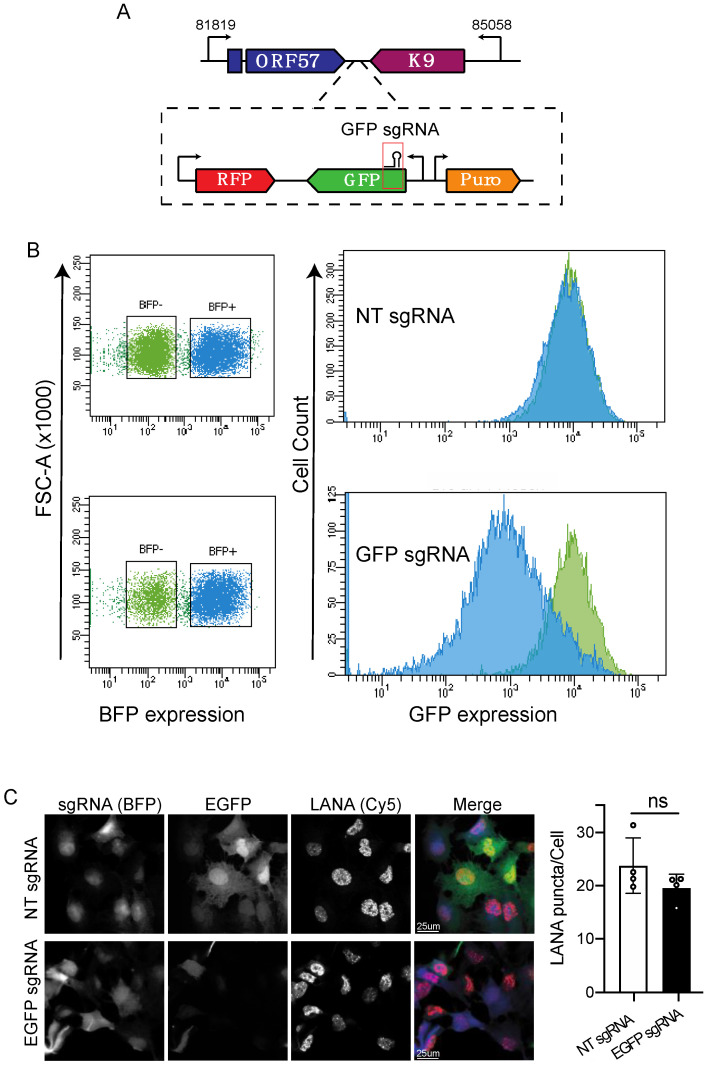
CRISPRi silencing of KSHV-encoded EGFP. (**A**) Region of the viral genome encoding EGFP and position of the sgRNA. (**B**) Flow cytometry analysis (MFI) of iSLK-219-dC9K cells transduced with sgRNAs targeting EGFP or non-targeting (NT) sgRNAs. Note the downregulation of EGFP expression in cells transduced with the targeting sgRNA. (**C**) Immunofluorescence and confocal microscopy analyses of paraformaldehyde-fixed, BFP expressing cells (sgRNA), EGFP and LANA in iSLK-219-dC9K cells transduced with sgRNAs targeting EGFP or non-targeting (NT) sgRNAs. Quantification of LANA puncta/Cell in four independent fields (*t*-Test, ns *p* = 0.15).

Next, we evaluated the efficiency of CRISPRi for silencing viral genes. We reasoned that the multiple copies of KSHV episomes could reduce the inhibitory effect of the CRISPRi machinery on viral genes by promoter competition and stoichiometry. To test whether CRISPRi can overcome the constraint imposed by viral gene expression derived from multiple transcriptionally active episomes, we transduced iSLK-219 cells with sgRNAs targeting the transcription start site (TSS) of the EGFP reporter gene encoded in the KSHV.219 genome (Figure 2A; Table S1). We chose EGFP to evaluate CRISPRi silencing because it is ubiquitously expressed in latently infected cells under the control of a constitutive EF-1 promoter, and it allows for the straightforward monitoring of changes in protein levels. Using flow cytometry, we detected sgRNA/BFP expression in greater than 80% of the transduced cells (Figure 2B), and we evaluated the levels of EGFP expression in unselected/unsorted cells. We found that the iSLK-219-dC9K cells expressing the targeting sgRNA (BFP^+^) have lower levels of EGFP when compared to non-transduced (BFP^−^) cells (Figure 2B). To confirm that the reduction in EGFP levels in sgRNA transduced cells was specific and not the result of episome loss, we immunostained for the latency-associated nuclear antigen (LANA), a viral protein required for the maintenance of latent infection. In these experiments, we observed that transduced cells showed low levels of EGFP, as well as the characteristic LANA punctate pattern that is associated with latent KSHV infection (Figure 2C). This observation indicates that the viral genome is still present and that LANA expression was unaltered after EGFP silencing. Together, these results demonstrate that CRISPRi is effective for silencing viral genes and is not hampered by the 30–100 copies of the KSHV genome present in infected cells.

### 3.2. CRISPRi Represses Latent and Lytic KSHV Genes and Curtails Infectivity

**Figure 3:**
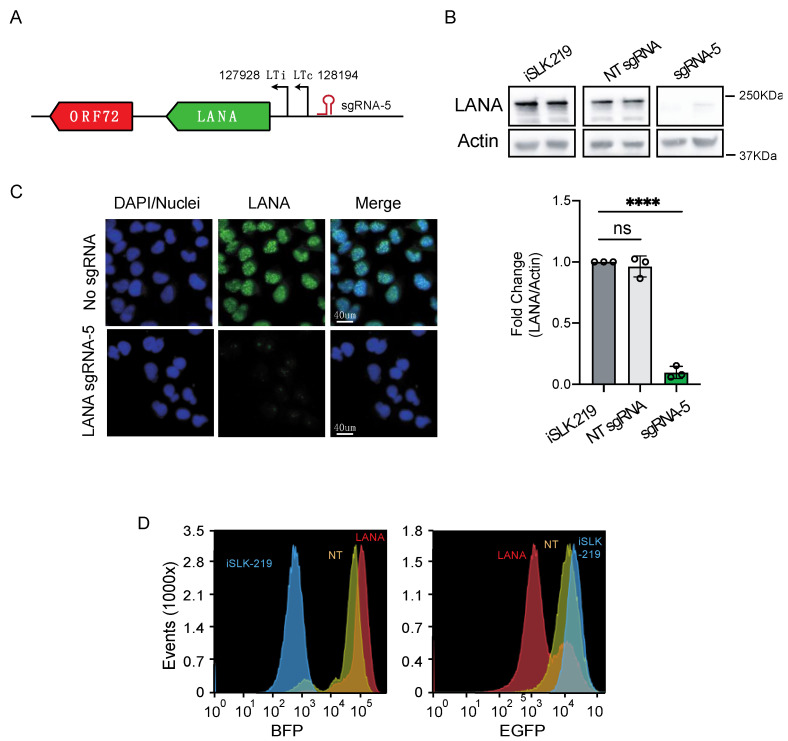
CRISPRi silencing of latent genes in iSLK-219 cells. (**A**) Region of the viral genome encoding LANA (ORF73) and position of the sgRNA. (**B**) Immunoblot analysis of LANA in iSLK-219 cells, and iSLK-219-dC9K cells transduced with a non-targeting (NT) sgRNA or a LANA specific sgRNA. Image representative of three biological replicates. Bars: quantification of the data. (one-way ANOVA, **** *p* < 0.0001). Error bars: standard deviation, ns, not significant. (**C**) Immunofluorescence analyses of LANA in methanol-fixed iSLK-219-dC9K cells transduced with a LANA specific sgRNA. Image representative of three biological replicates. (**D**) Flow cytometry analyses (MFI) of iSLK-219 (blue trace), or iSLK-219-dC9K cells transduced with a non-targeting (NT) sgRNA (yellow trace) or a LANA specific sgRNA (red trace). Note the downregulation of EGFP expression in cells where LANA has been silenced. Image representative of two biological replicates.

The viral cycle is divided into two main phases: latency, a persistent and dormant state with low viral gene expression, and the lytic cycle, a transcriptionally and translationally active state and the productive stage of infection. Having determined that CRISPRi can effectively repress viral-encoded genes (EGFP, Figure 2B), we next examined whether CRISPRi is equally effective at silencing latent and lytic genes. First, we explored CRISPRi silencing of a KSHV latent gene. To this end, we chose to silence LANA, a key mediator of latent viral replication that tethers the viral episome to host chromosomes by bridging the interaction between the KSHV genome and host chromatin [40,41,42]. To test whether CRISPRi repressed LANA, we transduced iSLK-219-dC9K cells with sgRNAs targeting the LTc promoter (Table S2, Figure 3A), which is known to be preferentially used for LANA transcription in these cells [6]. Two weeks after transduction, we selected the upper quartile of BFP^+^/sgRNA^+^ expressing cells by FACS and evaluated LANA levels in this population. We observed a substantial reduction in LANA expression by immunofluorescence and immunoblot analyses (~90% expression loss, *p* < 0.0001) (Figure 3B,C) and a concomitant reduction in the expression of the virus-encoded EGFP reporter by flow cytometry in cells transduced with LANA-targeting sgRNAs (Figure 3D). These results indicate that CRISPRi loss-of-function of LANA results in the loss of viral episomes and agree with recent observations showing that CRISPR-Cas9-mediated mutagenesis of LANA results in episome loss and latency disruption [18]. Together, these results indicate that CRISPRi can mediate the efficient knockdown of KSHV latent genes.

**Figure 4:**
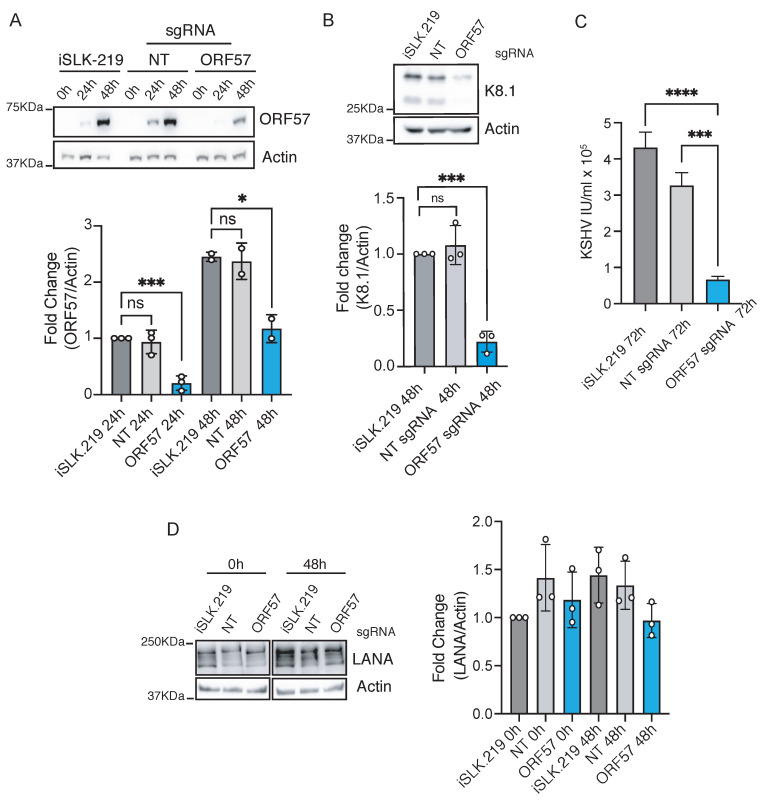
CRISPRi silencing of immediate–early genes in iSLK-219 cells. Immunoblot analysis of (**A**) ORF57 (one-way ANOVA, 24 h **** *p* < 0.001, 48 h * *p* value 0.025), (**B**) K8.1 (one-way ANOVA, 48 h *** *p* < 0.001) and (**D**) LANA (Kruskal–Wallis test, 0.99 < *p* < 0.3) in latent and lytic (24 and 48 h post-reactivation) iSLK-219 cells, and iSLK-219-dC9K cells transduced with a non-targeting (NT) sgRNA or an ORF57-specific sgRNA. Image representative of three biological replicates. Bars: quantification of the data. Error bars: standard deviation, ns, not significant. (**C**) Quantification of viral titers (IU, infectious units) in the filtered supernatant of iSLK-219 or iSLK-219-dC9K cells transduced with a non-targeting (NT) sgRNA or an ORF57-specific sgRNA, collected at 72 h post-reactivation. Error bars: standard deviation. (one-way ANOVA, **** *p* < 0.001, *** *p* < 0.001).

Next, we determined the ability of CRISPRi to silence lytic genes. KSHV lytic gene expression is classified into three stages based on their kinetics: immediate early, in which the first genes expressed upon entry to the lytic cycle are observed; delayed early, characterized by the expression of genes before DNA replication; and late, in which genes are expressed after DNA replication [43]. We first examined the CRISPRi-mediated silencing of the immediate early lytic gene ORF57, also known as mRNA transcript accumulation (MTA). ORF57 plays a crucial role in viral gene expression by enhancing RNA stability and splicing and promoting protein synthesis. Consequently, ORF57 deletion, silencing, or inactivation reduces viral reactivation and replication [44,45]. To silence ORF57, we transduced iSLK-219-dC9K cells with a sgRNA targeting the promoter at the annotated TSS (−3 to +15 bp) (Table S2, Figure 4A). Ten days after transduction, we selected the upper quartile of cells based on BFP^+^/sgRNA expression levels by FACS. We prompted entry into the lytic cycle in this population by exogenous RTA expression induced by doxycycline treatment [36]. We evaluated the expression of ORF57 at 0 h (latent), 24 h (early lytic), and 48 h (late lytic) post-reactivation and found significant downregulation (~90% knockdown, *p* < 0.001) at 24 h post-reactivation. The potency of CRISPRi inhibition was lower yet statistically significant at 48 h post-reactivation (~50% knockdown, *p* = 0.025), possibly due to an increase in the number of viral genomes following viral DNA replication and dilution of the repressive effect of dC9K. The silencing of ORF57, even when partial at 48 h, caused a substantial reduction in the expression of viral lytic genes, including K8.1, a canonical lytic marker (~86% reduction, *p* < 0.001) (Figure 4B), and a drop in viral replication (80–85% reduction in viral titers, *p* < 0.0001) (Figure 4C), without any measurable impact on latent gene expression (4D). Together, these results indicate that CRISPRi can efficiently silence immediate–early lytic KSHV genes.

**Figure 5:**
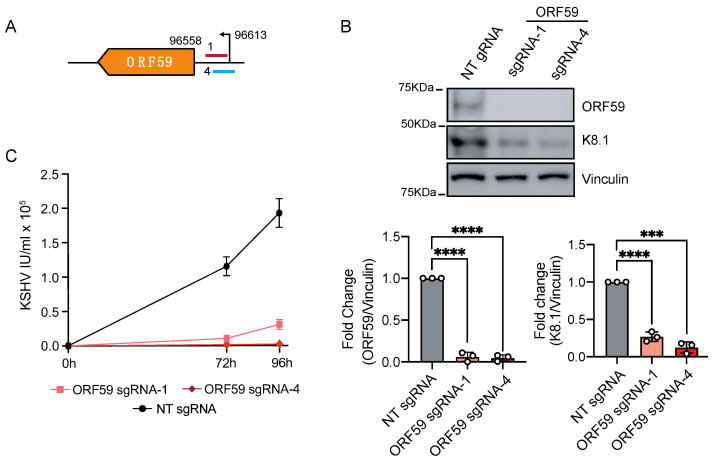
CRISPRi silencing of delayed-early genes in iSLK-219 cells. (**A**) Region of the viral genome encoding ORF59 and position of the sgRNAs. Immunoblot analysis of (**A**) ORF59, and (**B**) K8.1 (in latent and lytic (96 h post-reactivation) in iSLK-219-dC9K cells transduced with a non-targeting (NT) sgRNA or an ORF59-specific sgRNA. Image representative of three biological replicates. Bars: quantification of the data. Error bars: standard deviation (one-way ANOVA, **** *p* < 0.001, *** *p* < 0.001). (**C**) Quantification of viral titers (IU, infectious units) in the filtered supernatant of iSLK-219-dC9K cells transduced with a non-targeting (NT) sgRNA or ORF59-specific sgRNAs, collected at 72 and 96 h post-reactivation. Error bars: standard deviation.

Motivated by these findings, we next investigated whether CRISPRi can repress the transcription of KSHV delayed-early lytic genes. To this end, we focused on the promoter of ORF59, a viral processivity factor that coordinates the translocation of the viral polymerase (ORF9) to the nucleus. The ablation of ORF59 disrupts DNA replication and impedes virion production [46,47,48]. We targeted the promoter of ORF59 with two sgRNAs in the proximal region of the TSS (+24 bp from TSS on the template strand and +14 bp from TSS on the coding strand) (Table S2, Figure 5A). Ten days after transduction, we selected the upper quartile of cells expressing BFP^+^/sgRNA^+^ by FACS and induced entry to the lytic cycle by doxycycline-driven exogenous expression of RTA. After 96 h had passed following reactivation, we evaluated the expression of ORF59 by immunoblot and found a substantial downregulation of ORF59 (>90% reduction, *p* < 0.0001) (Figure 5B). As anticipated, and due to the critical function of ORF59 during the lytic cycle, the silencing of ORF59 resulted in the loss of late lytic proteins, including K8.1 (73–75% reduction, *p* < 0.0001) (Figure 5B), and a concomitant decrease in virus replication (80–90% reduction in titers) (Figure 5C). Our results show that CRISPRi efficiently represses delayed early lytic genes.

### 3.3. CRISPRi Represses Viral Genes in PEL-Derived Cells

**Figure 6:**
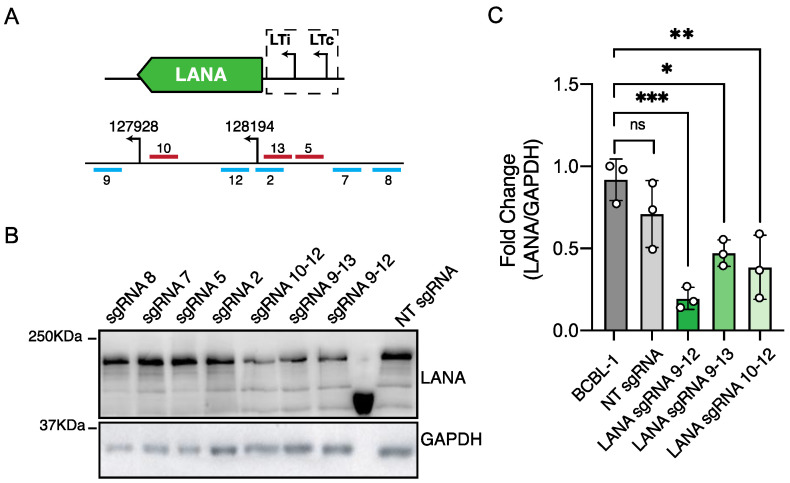
CRISPRi silencing of latent genes in BCBL-1 cells. (**A**) Region of the viral genome encoding LANA (ORF73) and position of the sgRNAs. (**B**) Immunoblot analysis of LANA in iSLK-219 cells, and iSLK-219-dC9K cells transduced with a non-targeting (NT) sgRNA or a LANA specific sgRNA. Image representative of three biological replicates. (**C**) Quantification of the immunoblot data for sgRNAs that silenced LANA. Error bars: standard deviation (one-way ANOVA, *** *p =* 0.0004, ** *p* = 0.004, * *p* = 0.0124, ns, not significant).

The results obtained in iSLK-219-dC9K cells (Figure 2, Figure 3, Figure 4 and Figure 5) indicated that CRISPRi silences KSHV genes belonging to different kinetic classes. Since the host cell type can significantly affect the choice of viral promoters and the regulation of viral gene expression in KSHV-infected cell lines [6], we sought to determine the efficiency of CRISPRi in BCBL-1 cells. To this end, we transduced BCBL-1-dC9K cells with sgRNAs targeting the LANA promoter (Table S2). BCBL-1 cells regulate LANA expression through two different promoters LTc and LTi [6,49]. We noted no gene expression changes ten days after transduction when we used the same sgRNA that we successfully used in iSLK-219-dC9K cells, sgRNA-LANA-5 (Figure 6B). Neither did we observe any measurable changes in gene expression when we targeted several other sgRNAs to different regions around the LANA LTc promoter (Figure 6B). We reasoned that an alternative promoter choice may compensate for LANA expression in these conditions, so we simultaneously targeted both LANA promoters in BCBL-1-dC9K cells with two sgRNAs complementary to the TSS at the LTc and the LTi promoters (Figure 6A). Using this approach, we observed the significant repression of LANA expression (50–80% reduction, *p* < 0.001–0.01) in BCBL-1 cells (Figure 6B,C). We achieved the best silencing when we positioned the sgRNAs immediately downstream of the LTc (17+ to 27+ bp) and the LTi (21+ to 30+ bp) LANA promoters. Together, these results demonstrate that CRISPRi effectively silences KSHV genes in different cellular backgrounds.

## 4. Discussion

CRISPR-based technologies have transformed our ability to conduct functional genomics studies. Two modifications of the CRISPR-Cas9 system, CRISPR activation (CRISPRa) and CRISPR interference (CRISPRi), permit manipulating gene expression with unprecedented precision and have opened up new ways for functional gene characterization. CRISPRi has been extensively used in mammalian, plant, and bacterial cells for single-gene and genome-wide transcriptional repression [26,27,29,30,31]. A recent study by Hein and Weissman highlights the advantages of CRISPRi for the genome-wide study of virus–host interactions, as it allows one to interrogate the role of essential host genes in infection, a feature commonly missed by CRISPR-Cas9 screens, and opens the possibility for interrogating viral gene functions [50]. Here, we show that CRISPRi can be used to repress the expression of latent and lytic KSHV genes, and that these manipulations curtail infectivity in cells in culture. Our results provide proof-of-concept for the use of CRISPRi to characterize viral gene functions.

CRISPRi offers two key advantages over CRISPR-Cas9 gene editing (knockouts) to study viral gene function. First, CRISPRi does not cause dsDNA breaks and it does not rely on the host cell DNA repair machinery, which circumvents the problem of generating different mutations in the multiple copies of the viral genome present in infected cells. Second, CRISPRi can access promoter elements in all episomes, thus obviating the need for targeting every single viral locus to achieve penetrant loss-of-function. Our results show that CRISPRi can be recruited to and repress promoters of latent and lytic genes in the multiple KSHV genomes present in these cells. These characteristics make CRISPRi an ideal technology to modulate gene expression in cells infected with herpesviruses.

It is noteworthy that the efficiency of CRISPRi for targeting specific loci seems to depend on the timing of the viral life cycle. For instance, the efficient silencing of ORF57 dwindled as the lytic cycle progressed. It is possible that reduction in the repressive effect of CRISPRi on ORF57 at late timepoints following reactivation is due to the replication of viral DNA and the accumulation of viral genomes, and the saturation of the available dC9K. This notion is supported by our observation of the sustained silencing of ORF59, a delayed-early gene, at 96 h post reactivation. The ablation of ORF59 function prevents viral DNA replication, and thus, under these conditions, the number of genomic targets for dC9K remain constant. Further optimization of the CRISPRi system in KSHV-infected cells, including the selection of pseudoclonal populations with high levels of dC9K expression, may produce more robust silencing of lytic genes following genome replication. Even without such optimizations, we note that the subdued repression of ORF57 at 48 h post-reactivation still resulted in impaired viral replication.

The inhibitory effect of CRISPRi is largely affected by the selection of the targeting sgRNA [51]. CRISPRi is most effective when the sgRNA targets the region −50/150+ bp from the TSS, with optimum activity in the region between 0/50+ from the TSS [27,51]. Our results corroborate these observations and show that the selection and position of the sgRNA is critical to tune the efficiency of gene repression of target viral genes. The strong silencing of LANA, ORF57, and ORF59 we achieved demonstrates that CRISPRi can approximate viral gene expression levels similar to those seen with loss-of-function approaches. CRISPRi also allows for the interrogation of the function of viral elements that cannot be targeted by RNAi or CRISPR knockouts, including nuclear RNAs and small and long non-coding RNAs, many of which remain uncharacterized in KSHV.

Another strength of CRISPRi for functional genomics studies of virus infected cells is its ability to grade gene expression and protein abundance. As opposed to true knockouts, partial gene loss-of-function can be useful for viral biology studies as it may allow for the bypassing of adaptation effects associated with complete loss-of-function upon gene editing. The careful design of gRNAs could be used to generate hypomorphic alleles by CRISPRi to titrate viral gene expression. Moreover, allelic series based on the expression of different levels of dC9K-sgRNA pairs can be established by selecting pseudoclonal populations. Such populations would allow one to study the effects of viral gene product dosing on infectivity.

The differences we observed in the repression of LANA when using the same sgRNAs in iSLK-219 or BCBL-1 cells may reflect the abundance of viral genomes in epithelial vs. B-cells. BCBL-1 cells have higher numbers of KSHV genomes that could lead to the saturation of available dC9K in these cells, resulting in the reduced efficiency of viral gene silencing by CRISPRi. The cell-type-specific regulation of the viral epigenome offers an alternative explanation for the different efficiency of CRISPRi in repressing viral gene expression in either cellular background. Indeed, recent studies on the genome-wide structure and regulation of viral chromatin, and the detailed annotation of the KSHV transcriptome, show that the cellular context has an impact on viral gene expression and transcriptional control. In the particular case of LANA transcription, two promoters, the constitutive LTc and the RTA-responsive LTi, have been identified [52]. A recently detailed annotation of viral transcription start sites in iSLK-219 and BCBL-1 cells show that LTc is used for LANA transcription in both cellular contexts, while LTi is exclusively used in BCBL-1 cells [6]. The factors that determine the selection and use of LTi in different cell lines remain to be determined. These observations underscore the importance of obtaining detailed viral epigenomes and transcriptomes to inform sgRNA design for efficient CRISPRi of viral genes. Another aspect that needs to be carefully considered for viral gene CRISPRi is the compact nature and high density of coding elements of viral genomes, which could increase off-target effects. While CRISPRi has been shown to have minimal off-target effects on cellular genes [26], it will be crucial to determine off-target effects when targeting viral genes, particularly those encoded by polycistronic transcripts, and those for which transcription is controlled by bidirectional promoters or promoters with overlapping regulatory elements.

Finally, combining the strengths of CRISPRi with CRISPRa will enable comprehensive functional genomics studies in KSHV and other viruses by providing complementary biological insights. Similar to CRISPRi, CRISPRa targets endogenous promoters to activate genes by inducing their transcription at near-physiological levels, which is often difficult to achieve through ectopic overexpression. A recent report by Elbasani et al. demonstrated the successful activation of the viral transcription factor RTA in KSHV-infected cells, setting the precedent for the use of CRISPRa in viral gene gain-of-function studies [53]. Together, these new technologies will enable the discovery of the characterization of viral features, and guide new biological models in virology.

## Supplementary Materials

The following are available online at https://www.mdpi.com/article/10.3390/v13050783/s1, Table S1: sequence of sgRNAs targeting cellular genes and controls, Table S2: sequences and genomic coordinates of sgRNAs targeting KSHV genes.
