# Supplementary material for: CRISPR Interference Efficiently Silences Latent and Lytic Viral Genes in Kaposi’s Sarcoma-Associated Herpesvirus-Infected Cells
Source: Viruses. 2021 Apr 28;13(5):783. doi: 10.3390/v13050783 (PMC8146339; doi:10.3390/v13050783)
Supplement: Supplementary file 1 [file viruses-13-00783-s001.zip › viruses-1193826-supplementary.pdf]

Other sgRNAs

Target Gene	Protospacer
**GFP-T1 sgRNA**	GGGCGAGGAGCTGTTCACCG
**ATF6 sgRNA**	GTTAATATCTGGGACGGCGG
**PERK sgRNA**	GACAGCCAGCCGTGTTCCCC
**Non-targeting (Gal4) sgRNA**	GAACGACTAGTTAGGCGTGTA

Table S1: Sequence of sgRNAs targeting cellular genes and controls

Target Gene	Target Strand	gRNA start	gRNA end	Distance from TSS (bp)			Protospacer
				iSLK.219	BCBL-1 LTi	BCBL-1 LTc	
**LANA sgRNA-2**	-	128217	128235	4	289	23	GACACATTTTTTGATTGTCC
**LANA sgRNA-5**	+	128257	128238	44	329	63	GCCCTCCACTACGCGGCGCC
**LANA sgRNA-7**	-	128354	128373	141	426	160	GTGCTCCCGGTTGCTGGCAC
**LANA sgRNA-8**	+	128428	128409	215	500	234	GGCACTCGGCGTCGTCCACG
**LANA sgRNA-9**	-	127901	127919	312	27	293	GAAGGTTAAAGTGGGTTTGCG
**LANA sgRNA-10**	+	127945	127926	268	17	249	GTAGCATCCATGGCAACGCAG
**LANA sgRNA-12**	-	128173	128191	40	245	21	GTGGGGACGTGACTGCTTCG
**LANA sgRNA-13**	+	128224	128206	11	296	30	GAAATGTGTGTATCATTTGG
**ORF57 sgRNA**	+	81834	81816	15	N/A	N/A	GCGTATGCCGCGGGCTATTT
**ORF59 sgRNA-1**	+	96589	96570	24	N/A	N/A	GTGATTTCTCTGTGCGTCTA
**ORF59 sgRNA-4**	-	96599	96617	14	N/A	N/A	GGCGCGCACACTGGGTGTTC

Table S2: Sequences and genomic coordinates of sgRNAs targeting KSHV genes
